# Respiratory function after 30+ years following sulfur mustard exposure in survivors in Sweden

**DOI:** 10.3389/fmed.2024.1251500

**Published:** 2024-03-04

**Authors:** Faraidoun Moradi, Sanna Kjellberg, Ying Li, Bledar Daka, Anna-Carin Olin

**Affiliations:** ^1^Department of Public Health and Community Medicine, Institute of Medicine, Sahlgrenska Academy, University of Gothenburg, Gothenburg, Sweden; ^2^The Centre for Disaster Medicine, University of Gothenburg, Gothenburg, Sweden

**Keywords:** small airways, impulse oscillometry, multiple breath washout, pulmonary disease, sulfur mustard, Halabja

## Abstract

**Background:**

Sulfur mustard (SM) exposure causes acute and chronic respiratory diseases. The extent of small airway dysfunction (SAD) in individuals exposed to SM is unclear. This study evaluated and compared SAD in SM-exposed and SM-unexposed participants using noninvasive lung function tests assessing small airway function.

**Methods:**

This retrospective cohort study involved SM-exposed (*n* = 15, mean age: 53 ± 8 years) and SM-unexposed (*n* = 15, mean age: 53 ± 7 years) Kurdish-Swedish individuals in Sweden. Small airway resistance and reactance were assessed using impulse oscillometry (IOS). Nitrogen (N_2_) multiple breath washout (MBW) was employed to assess lung ventilation heterogeneity. The gas-exchanging capacity of the lungs was assessed using the diffusing capacity of the lungs for the carbon monoxide (DLCO) test. Lung function outcomes were reported as absolute values and *z*-scores. Group comparisons were performed using the Mann–Whitney U test.

**Results:**

No statistically significant differences in age, height, or body mass index were observed between the two groups. IOS showed significantly increased small airway resistance, while N_2_MBW exhibited significantly increased global and acinar ventilation heterogeneity in SM-exposed individuals compared to that in unexposed individuals. SAD was identified in 14 of 15 SM-exposed individuals, defined as at least one abnormal IOS difference between resistance at 5 and 20 Hz (R5-R20) and/or area of reactance (AX) or N_2_MBW lung’s acinar zone (S_*acin*_), and DLCO adjusted to the alveolar volume (DLCO/VA) outcome. Of these 14 individuals, only 5 demonstrated concordant findings across the IOS and N_2_MBW tests.

**Conclusion:**

Exposure to SM was positively associated with long-term impairment of respiratory tract function in the small airways in the majority of the previously SM-exposed individuals in the present study. Furthermore, both IOS and N_2_MBW should be employed to detect SAD in SM-exposed survivors as they provide complementary information. Identifying and characterizing the remaining pathology of the small airways in survivors of SM exposure is a first step toward improved treatment and follow-up.

## 1 Introduction

Sulfur mustard (SM) is a blistering agent with alkylation capacity and lipophilic ability to form a highly reactive intermediate cyclic sulfonium ion, which can bind to many biological molecules (1–3). Details of its underlying pathological and biochemical mechanisms of action are unclear; hence, no antidotes or specific medications have been identified that treat or delay respiratory symptom progression (4).

The short and long-term effects of SM exposure primarily affect the eyes, skin, and lungs (5, 6). Over 80% of SM-exposed survivors reportedly develop respiratory symptoms, which are identified as the leading cause of mortality in survivors post-exposure (7–9).

One significant long-term consequence of SM exposure is bronchiolitis obliterans (10, 11). Other long-term effects include varying severe symptoms, such as hypersensitivity reactions, chronic dyspnea, recurrent pneumonia, chronic bronchitis, and chronic obstructive pulmonary disease (COPD) (12–15). Imaging and clinical investigations have revealed various pulmonary injuries, such as bronchiectasis, atelectasis, mosaic parenchymal attenuation, irregular and dilated central airways, bronchial wall thickening, interlobular septal wall thickening, pulmonary fibrosis, cryptogenic organizing pneumonia, emphysema, tracheomalacia, airways stenosis, and cancer, in both large and small airways (12–17).

The pulmonary pathological manifestations of exposure to SM may deteriorate over time, with individuals exposed to SM who were neither evacuated nor hospitalized after the exposure, being at risk of developing respiratory complications several decades later (9). Therefore, it is crucial to monitor individuals at high risk for any potential respiratory problems arising from SM exposure and employ alternative non-traditional monitoring methods to identify early-stage respiratory issues. While spirometry tests are the gold standard for measuring lung function, they have demonstrated a mixed picture of asthma, chronic obstructive pulmonary disease, and even normal results in SM-exposed survivors (18–21). Owing to the limited resources, conducting high-quality spirometry in many participants proved challenging; therefore, it was excluded from the study protocol in this article. Moreover, spirometry tests register modifications in airways in symptomatic patients and may not detect early-stage respiratory issues; further, they are not sensitive enough to measure the function of the small airways (airways with an internal diameter <2 mm) until significant clinical symptoms occur (22–24). Small airway dysfunction (SAD) is a major pathological aspect of lung illness, including bronchiolitis obliterans in SM-exposed survivors (25) and in patients with asthma and COPD (13, 26).

Assessing SAD can be challenging as no noninvasive gold standard is currently available (22). Spirometry, although most commonly employed, lacks consensus on the best parameter or criteria for identifying SAD. Forced expiratory flow at 25% and 75% of the pulmonary volume (FEF 25–75) is the most widely used parameter. However, its reproducibility is limited by its dependence on the forced expiratory volume in 1 s (FEV1)/forced vital capacity (FVC) and lack of adjustment for lung volume (27–29). Thus, the spirometry test is considered less sensitive in detecting modifications in small airways in the early stage; however, it may detect mild lung injury (23, 30, 31). Despite normal spirometry test results, biopsy findings indicated obliterative bronchiolitis in the small airways of the lungs in 50% of the patients 20 years after exposure to SM (10). However, spirometry tests for identifying SAD is questionable, warranting further research (32, 33). Chest high-resolution computed tomography (HRCT) is another noninvasive method that has been used for studying and evaluating abnormal changes in the morphology of small airways; however, its repeated use is limited owing to radiation risks (34). Nevertheless, follow-up assessments should be conducted in SM-exposed patients with SAD to determine the effectiveness of therapeutic interventions.

Hence, alternative lung function methods are warranted to non-invasively identify early SAD in patients, allowing timely follow up and early interventions in case of clinical respiratory manifestations. Previous studies have suggested that impulse oscillometry (IOS) and nitrogen (N_2_) multiple breath washout (MBW) may exhibit greater sensitivity in identifying early-stage SAD than spirometry (23, 30, 35). IOS and N_2_MBW are noninvasive physiological lung tests that measure different aspects of small airway function. IOS measures the mechanical properties of the lung (i.e., resistance and reactance) (36), while N_2_MBW provides information about ventilation distribution within the lung as a whole (lung clearance index, LCI) and more distally by using the indices of ventilation heterogeneity in the conducting (S_*cond*_) and acinar airways (S_*acin*_), which are indicators of the rate of nitrogen washout (24, 37). Moreover, the gas exchange capacity in the lung across the alveolar-capillary interface can be assessed using the diffusing capacity of the lungs for the carbon monoxide (DLCO) test (38). The DLCO was employed to examine whether exposure to SM could impact this region of the lung in long-term. SAD has not been thoroughly studied in individuals exposed to SM with other non-traditional, noninvasive pulmonary function tests (PFTs). This study aimed to compare the outcomes of different noninvasive PFTs, e.g., IOS, N_2_MBW, and DLCO, to assess small airways, including alveolar function in SM-exposed survivors. We further compared these results with those of individuals who were not exposed to SM from the same ethnic group.

## 2 Materials and methods

This study evaluated data from a retrospective cohort study of participants exposed to SM from 1987 to 1988 in the Kurdistan Regions of Iraq and Iran. Participants recruited from Kurdish-Swedish survivors resettled in Sweden (exposed) were compared with SM-unexposed first-generation Kurdish-Swedish citizens (unexposed), ensuring that the sociodemographic characteristics were as similar as possible in both groups. Participants were recruited using posters about the study, social media announcements, and word of mouth.

The inclusion criteria for the SM-exposed group were as follows: (i) persons originally from the provenance of SM-attacked areas in the Kurdistan regions of Iraq and Iran who survived the chemical attacks of 1987 and 1988, (ii) persons with physical symptoms that developed at the time of SM exposure (signs of SM exposure), and (iii) persons aged 34–80 years. The inclusion criteria for the SM-unexposed group were as follows: (i) persons originally from Kurdistan, (ii) persons with no history of SM exposure, and (iii) persons aged 34–80 years. According to the study protocol, each exposed participant was matched with one unexposed participant. Data from 30 participants (exposed vs. unexposed: IOS, 15 vs. 15; N_2_MBW, 15 vs. 14; and DLCO, 13 vs. 14) were collected and included in the current study.

All the participants provided written informed consent prior to inclusion in the study. Data from five unexposed participants and one SM-exposed participant were collected between October and November 2019, and the remaining data were collected between March and June 2022. Data collection ceased during the coronavirus disease pandemic. The Regional Ethical Review Board of Gothenburg, Sweden, approved this study (2017-12-07599-17).

### 2.1 Exposure assessment

During the Iran-Iraq War in the 1980s, the Kurdish cities of Halabja and Sardasht were heavily attacked using chemical warfare agents, such as SM (39–41). There is no objective confirmation of SM exposure owing to a lack of evidence-based laboratory tests to verify exposure to SM after so many years. However, the exposure history was determined based on the anamnesis of the exposed participants, who originally came from chemical warfare agent-attacked regions in Kurdistan in Iraq and Iran. It is worth noting that survivors of the Halabja chemical attack faced challenges in receiving initial medical care due to limited support and delayed access to aid by the Iraqi regime. Despite some individuals receiving assistance in Iran, not all could escape or receive urgent treatment, resulting in a lack of medical documentation and verification of their exposure. *Primary exposure* was defined as direct contact with or inhalation of SM owing to an explosion of nearby bombs. *Secondary exposure* involved indirect contact with contaminated bodies or objects following the chemical attacks.

### 2.2 Pulmonary function measurements

Before the lung investigations, SM-exposed participants completed a questionnaire concerning the possible dose, duration, and route of SM exposure, initial signs and symptoms, current physical symptoms, medication, and other demographic variables. The IOS and N_2_MBW tests were conducted before the DLCO test to minimize the effect of forced expiration on airway tone; no bronchodilators were used during the tests. No coherence was used for quality control of IOS.

Impulse oscillometry was performed using the Jaeger Master Screen system (CareFusion, Germany) according to the current guidelines (42). A loudspeaker generated sound waves ranging from 5 to 35 Hz, which were transferred into the respiratory system via a suitable mouthpiece. Participants sat upright on a chair fitted with a nose clip and were asked to breathe normally through a mouthpiece for 30 s. To reduce the effect of “shunt-impedance,” the participant was asked to support their cheeks with their hands during the recordings. Mean values from three artifact-free recordings were reported. Results were acceptable if the coefficient of variation of at least 2 sets of data was less than 10%. The reported IOS variables included: (i) resistance at 5 Hz (R5) as a measure of airway resistance from the mouth to the distal point in the airway tree where the soundwave faded out, (ii) resistance at 20 Hz (R20) as a measure of central airway resistance, (iii) the difference between resistance at 5 and 20 Hz (R5-R20) as a measure of small airway resistance (43), (iv) area of reactance (AX), (v) reactance at 5 Hz (X5), both reflecting the elastance (stiffness) of the small airways, and (vi) resonant frequency (fres) reflecting the frequency at which total reactance is zero (44). All the IOS outcomes were reported in kPa/L/s (kPa/L for AX) and in *z*-scores (except for fres) based on the findings in a locally collected healthy control cohort (24), fres was measured in Hz (44).

Nitrogen multiple breath washout was measured using an ExhalyzerD device (EcoMedics, Switzerland) with the software Spiroware 3.3.1. Resident N_2_ was washed out from the lung by breathing 100% oxygen (O_2_). ExhalyzerD was validated (45) according to the current guidelines for inert gas washout tests. The study’s recordings were also conducted according to these guidelines; however, two artifact-free registrations deemed sufficient instead of three (46). Participants were instructed to wear a nose clip, sit straight with both feet flat on the floor, and breathe normally into a mouthpiece in a relaxed position. When a stable tidal breathing pattern was attained, the washout phase was initiated by breathing 100% oxygen. It was completed when the exhaled end-tidal N_2_ concentration was below 1/40th (2.5%) of its initial concentration. The reported outcomes included LCI 2.5%, S_*cond*_, and S_*acin*_. LCI 2.5%, which reflects global ventilation heterogeneity, is calculated by dividing the cumulative expired volume during the washout by functional residual capacity (FRC) obtained from the same washout curve. Thus, LCI 2.5% is an index of how often the lung volume (FRC) needs to be “turned over” to wash out resident N_2_ below 2.5% of the starting concentration. S_*cond*_ and S_*acin*_ are derived from a so-called concentration-normalized phase III slope (Sn_*III*_) analysis. These indices provide information about the location within the airway tree where ventilation heterogeneity is present. S_*cond*_ reflects ventilation heterogeneity in the conducting airways, while S_*acin*_ reflects ventilation heterogeneity close to or at the entrance to the acinar airway zone (37). The *z*-scores were calculated using two locally collected healthy control cohorts (24).

Diffusing capacity of the lungs for the carbon monoxide was measured using the single-breath technique with a Jaeger Master Screen system (CareFusion, Germany). The test procedure was performed according to the guidelines for the test (47), and the outcomes were adjusted based on the participant’s current hemoglobin level. The test procedure involved the following steps: (i) Seating the participant in a chair-fitted with a nose clip and instructing them to breathe calmly at a normal rate. (ii) Having the participant perform maximum exhalation before a rapid and full inspiration to total lung capacity. (iii) Finally, instructing the participant to hold their breath at total lung capacity for approximately 10 s, followed by exhalation. During full inspiration, the participants inhaled medical lung test gas (ICOMAS, Linde Healthcare, Sweden) containing 0.3% carbon monoxide, 0.3% methane, 0.3% acetylene, and 20.9% O_2_ balanced in N_2_. The reported outcomes were alveolar volume (VA), DLCO, and their ratio (DLCO/VA). The *z*-scores were calculated based on the Global Lung Function Initiative reference equations for DLCO (48).

### 2.3 Statistical analysis

All the continuous demographic variables are presented as mean (standard deviation, SD) and median (min; max), while categorical variables are reported using frequency. Lung function outcomes are expressed as absolute values and *z*-scores. Limits of normality were defined as a *z*-score of <−1.96 or >1.96. The differences between the exposed and unexposed groups were determined based on the median (min–max) and *z*-score. Group comparisons were performed using the Mann–Whitney U test owing to not normally distributed data and small sample size. The significance level was set at *P* < 0.05. All the statistical analyses were performed using SPSS, version 28 (IBM Corp., Armonk, NY, USA).

## 3 Results

The participants’ characteristics are summarized in Table 1. No statistically significant differences were observed in the age, height, or body mass index between the two groups. All the participants, except one in the exposed group, reported no current or previous smoking history. Table 2 demonstrates the exposed participants’ characteristics, e.g., physical symptoms following exposure, of which 12 participants reported respiratory symptoms in the acute phase, 5 participants reported receiving early treatment following SM exposure, 12 experienced delayed respiratory symptoms, 6 participants had physician-diagnosed COPD, 2 were diagnosed for asthma and 1 for bronchiectasis, and 8 participants reported experiencing dyspnea upon walking on smooth ground (Table 2). None of the participants in the current study was wearing the oxygen.

**TABLE 1:** Characteristics of the study participants; exposed vs. unexposed in IOS (*n* = 15 vs. 15), N_2_MBW (*n* = 15 vs. 14), and DLCO (*n* = 13 vs. 14).

Variables	Unexposed	Exposed	*P*-value
Sex, *n* (%)			1.00*
Female	5 (33%)	5 (33%)	
Male	10 (67%)	10 (67%)	
Age (years)			0.86
Mean ± SD	53 ± 7	53 ± 8	
Median (min–max)	54 (43–69)	54 (35–70)	
Height (cm)			0.49
Mean ± SD	169 ± 7	167 ± 13	
Median (min–max)	169 (157–180)	170 (145–189)	
BMI (kg/m^2^)			0.46
Mean ± SD	28 ± 2	29 ± 4	
Median (min–max)	28 (25–31)	30 (23–40)	
Smoking, *n* (%)			1.00*
Yes	0 (100%)	1 (7%)	
No	0 (100%)	14 (93%)	
Type of exposure, *n* (%)			<0.001^#^
Primary	0 (100%)	10 (67%)	
Secondary	0 (100%)	5 (33%)	

*N*, number of participants; SD, standard deviation; BMI, body mass index. For categorical variables, *n* (%) is presented; for continuous variables, mean ± SD/median (min–max) is presented. The symbol “*” based on Fisher exact test. The symbol “#” based on Chi-squared test.

**TABLE 2:** Characteristics of the sulfur mustard (SM)-exposed participants.

No.	Age	Sex	BMI	Smoking	Acute system symptom	Delayed system symptom	Dyspnea during walking (1 = yes, 0 = no)	Diagnosed by physician	Date of exposure	History of acute medical treatment (0 = no, 1 = yes)	Place of exposure
1	48	M	25	1	Dermatological	Respiratory	0	0	1987	0	Sardasht, Kurdistan-Iran
2	70	F	40	0	Dermatological	Respiratory	0	0	1988	1	Halabja, Kurdistan-Iraq
3	57	M	24	0	Dermatological, ocular, and respiratory	Dermatological and ocular	0	0	1988	0	Shanaxse, Kurdistan-Iraq
4	57	M	31	0	Dermatological and respiratory	Dermatological and respiratory	0	COPD	1988	0	Halabja, Kurdistan-Iraq
5	53	F	28	0	Respiratory	0	1	0	1988	0	Halabja, Kurdistan-Iraq
6	47	M	24	0	Dermatological, respiratory, and ocular	Respiratory	1	Bronchiectasis	1987	1	Sardasht, Kurdistan-Iran
7	58	M	25	0	Dermatological and respiratory	Respiratory and ocular	0	COPD	1986	0	Go tape, Kurdistan-Iraq
8	59	M	31	0	Respiratory	0	0	0	1988	0	Halabja, Kurdistan-Iraq
9	56	M	30	0	Dermatological, respiratory, and ocular	Dermatological, ocular, and respiratory	0	COPD	1988	1	Mawet, Kurdistan-Iraq
10	38	F	23	0	Dermatological and respiratory	Respiratory	1	COPD	1988	1	Halabja, Kurdistan-Iraq
11	48	F	32	0	Ocular and respiratory	Respiratory	1	COPD	1988	0	Halabja, Kurdistan-Iraq
12	50	F	32	0	Dermatological and respiratory	Respiratory	1	COPD	1988	0	Halabja, Kurdistan-Iraq
13	53	M	30	0	Dermatological and respiratory	Ocular and respiratory	1	0	1988	0	Badinan, Kurdistan-Iraq
14	52	M	26	0	Dermatological, respiratory, and ocular	Respiratory	1	Asthma	1988	1	Halabja, Kurdistan-Iraq
15	35	M	30	0	Dermatological	Respiratory	1	Asthma	1988	0	Halabja, Kurdistan-Iraq

BMI, body mass index; COPD, chronic obstructive pulmonary disease.

### 3.1 Pulmonary function measurement outcomes

**FIGURE 1:**
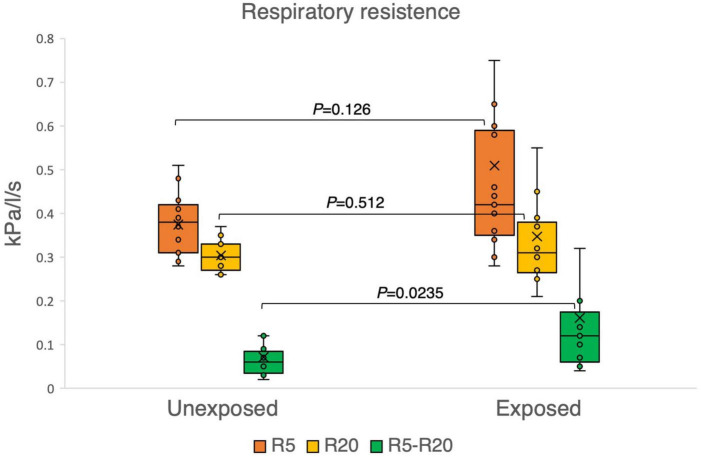
Outcomes of impulse oscillometry (IOS) variables demonstrating the respiratory resistance system total airway resistance (R5), central airways (R20), and peripheral small airways (R5-R20) in 15 exposed vs. 15 unexposed participants. *P*-value is based on independent samples Mann–Whitney U test. Box plots of IOS measurements: the boxes represent the 25th–75th percentiles with medians, and the top and bottom tails represent the highest/lowest scores without outliers. X shows the mean marker, and the circle shows the inner point.

**FIGURE 2:**
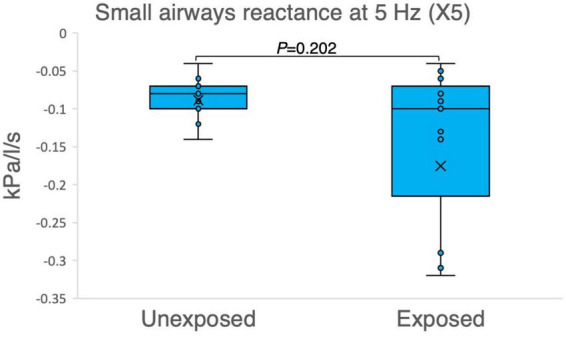
Outcomes of impulse oscillometry X5 (reactance at 5 Hz) in 15 sulfur mustard (SM)-exposed vs. 15 unexposed participants. *P*-value is based on independent-samples Mann–Whitney U test, used for between-group comparisons. Significance was set at *P* < 0.05.

**FIGURE 3:**
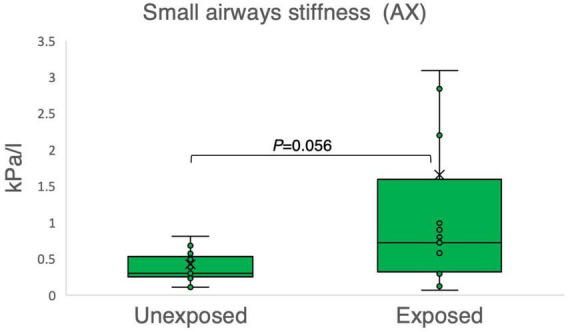
Outcomes of impulse oscillometry for the area of reactance (AX) in 15 sulfur mustard (SM)-exposed vs. 15 unexposed participants. *P*-value is based on independent-samples Mann–Whitney U test, used for between-group comparisons. Significance was set at *P* < 0.05.

**FIGURE 4:**
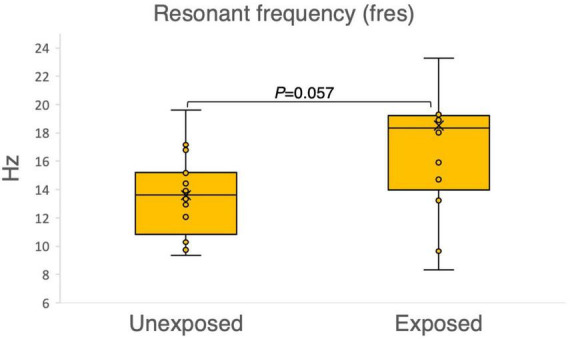
Outcomes of the impulse oscillometry resonant frequency (fres) in 15 sulfur mustard (SM)-exposed vs. 15 unexposed participants. *P*-value is based on independent-samples Mann–Whitney U test, used for between-group comparisons. Significance was set at *P* < 0.05.

The results of the variables derived from the IOS measurements provided insights into the respiratory function of both the exposed and unexposed groups, suggesting impaired function in the small airways for the exposed group. The exposed group showed significantly higher resistance in the peripheral small airways for measured value (R5-R20, *P* = 0.023) in Figure 1. On the other hand, Figures 2–4 depict the reactance of the respiratory system wherein the exposed group tended to have higher stiffness in the small airways (AX, *P* = 0.056), as the exposed group illustrated more negative reactance values (X5, *P* = 0.202) and higher resonant frequency (fres, *P* = 0.057) than the unexposed group. Nevertheless, the differences were not statistically significant.

**FIGURE 5:**
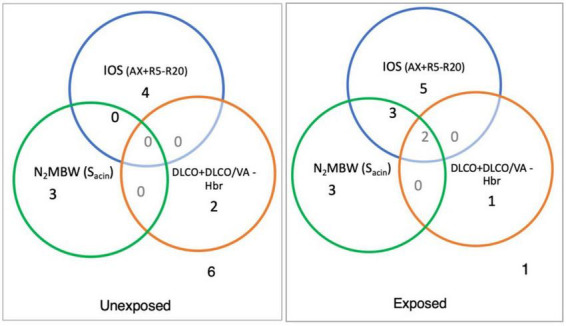
The number in each circle shows the number of abnormal values in the sulfur mustard (SM)-exposed and unexposed groups with different outcomes for specific variables for peripheral airways measurement with impulse oscillometry [R5-R20 and area of reactance (AX)), and nitrogen multiple breath washout method ventilation heterogeneity in the acinar airway zone (S_*acin*_) and the ratio of the diffusing capacity of the lung for carbon monoxide (DLCO) to the alveolar volume (DLCO/VA) separately or in combination (exposed vs. unexposed: IOS, 15 vs. 15; N_2_MBW, 15 vs. 14; and DLCO, 13 vs. 14).

Figure 5 illustrates the number of individuals with abnormalities in variables related to small airways for different techniques used in the study. The results revealed that among the exposed group, 10 of 15 individuals had abnormal values for IOS (AX and R5-R20), compared to 4 out of 15 individuals in the unexposed. Moreover, 8 out of 15 individuals in the exposed group and 3 out of 14 in the unexposed group had abnormal values for N_2_MBW (S_*acin*_). Lastly, the study found that 3 out of 13 individual and 2 out of 14 individuals had abnormal values for DLCO in exposed and unexposed group, respectively.

Based on the study findings, the prevalence of abnormalities varied greatly among participants when using different methods, such as IOS, N_2_MBW, and DLCO. The IOS method detected the highest number of abnormalities, while N_2_MBW showed a mix of abnormal results, and DLCO identified a lower number of abnormalities. Table 3 presents the details of the abnormalities found in the study population based on *z*-scores for various variables. Interestingly, the exposed individuals showed significantly more abnormalities than unexposed individuals in the IOS method variables R5 (6:0), R20 (3:0), and particularly in R5-R20 (8:0) and AX (10:4), which are specific to small airways.

**TABLE 3:** Abnormal values for variables based on *z*-score in the SM-exposed vs. unexposed groups; exposed vs. unexposed in IOS (*n* = 15 vs. 15), N_2_MBW (*n* = 15 vs. 14), and DLCO (*n* = 13 vs. 14).

Participants	R5	R20	R5-R20	AX	LCI 2.5%	S_*cond*_	S_*acin*_	IOS	N_2_MBW	DLCO	DLCO/VA
Unexposed	0	0	0	4	5	8	3	4	8	0	2
Exposed	6	3	8	10	14	11	8	10	14	1	2

AX, area of reactance; DLCO, diffusing capacity of the lung for carbon monoxide; VA, alveolar volume; IOS, impulse oscillometry; LCI 2.5%, lung clearance index; R5, resistance at 5 Hz; R20, resistance at 20 Hz; R5-R20, the difference between resistance at 5 and 20 Hz; N_2_MBW, nitrogen multiple breath washout; S_*acin*_, ventilation heterogeneity at the entrance to the acinar airway zone; S_*cond*_, ventilation heterogeneity in the conducting airways.

Similarly, using the N_2_MBW method, abnormalities were detected in the levels of LCI 2.5% (14:5), S_*cond*_ (11:8), and S_*acin*_ (8:3). Although S_*acin*_ represents the distal part of the small airways and exhibited noticeable differences, fewer differences were observed in S_*cond*_, which represents the central airways. Additionally, minor differences between the groups regarding DLCO (3:2) were noted.

**FIGURE 6:**
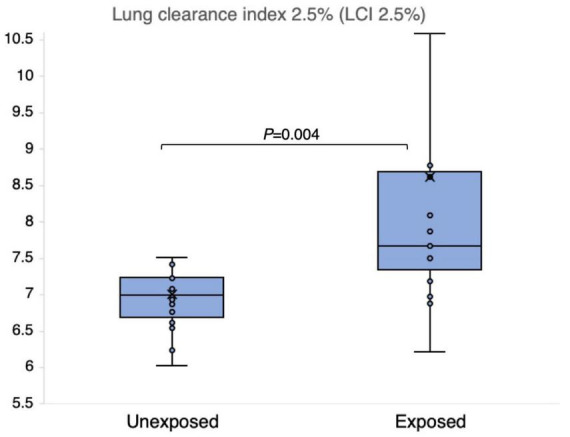
Outcomes of nitrogen multiple breath washout lung compliance index 2.5% (LCI 2.5%) in 15 sulfur mustard (SM)-exposed and 14 unexposed participants. *P*-value is based on independent-samples Mann–Whitney U test, used for between-group comparisons. Significance was set at *P* < 0.05.

**FIGURE 7:**
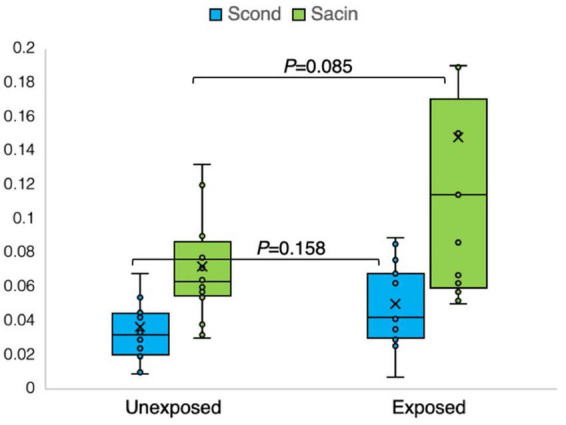
Outcomes of nitrogen multiple breath washout in the conducting (S_*cond*_) and acinar airway zone (S_*acin*_) in 15 sulfur mustard (SM)-exposed and 14 unexposed participants. *P*-value is based on independent-samples Mann–Whitney U test, used for between-group comparisons. Significance was set at *P* < 0.05.

The results of the N_2_MBW measurements revealed a more significant degree of global airway ventilation heterogeneity and a trend toward higher acinar ventilation heterogeneity in the exposed group than in the unexposed group (Figures 6, 7). The exposed group exhibited significant variation, with a few individuals displaying substantial deviations. Eight and three participants of the exposed and unexposed groups, respectively, had abnormal S_*acin*_ values (Figure 5). The overlap was even more pronounced for LCI, with 14 exposed participants and 4 unexposed participants surpassing the reference level (Table 3). However, LCI 2.5% and S_*acin*_ values of the exposed group differed significantly from those of the unexposed group (Table 3). In addition, the variation of S_*cond*_ within both the exposed and unexposed groups was not significant, with the presence of 11 and 8 individuals with S_*cond*_ abnormalities in the exposed and unexposed group, respectively (Table 3). Furthermore, no significant difference was observed in S_*cond*_ between the two groups (Figure 7).

**FIGURE 8:**
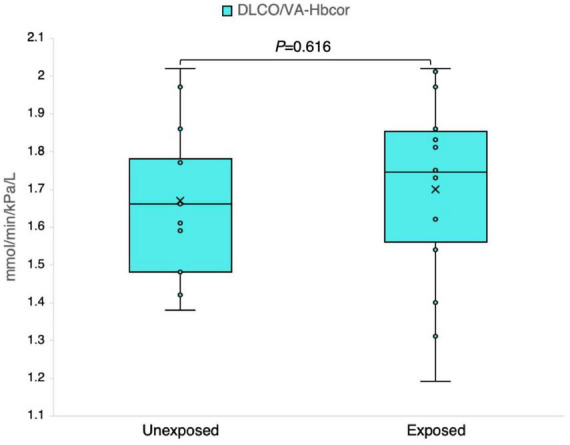
Outcomes of the ratio of the diffusing capacity of the lung for carbon monoxide (DLCO) to the alveolar volume (DLCO/VA) adjusted for the hemoglobin level in 13 sulfur mustard (SM)-exposed and 14 unexposed individuals. *P*-value is based on independent-samples Mann–Whitney U test, used for between-group comparisons. Significance was set at *P* < 0.05.

The results obtained from the analysis of the DLCO-derived variables demonstrated no significant impact on the studied groups; however, a few individuals demonstrated noteworthy abnormalities for the *z*-scores (Table 3). Figure 8 clearly illustrates no significant differences in the absolute measured values and *z*-scores of the DLCO/VA between the exposed and unexposed groups.

Based on the findings from the calculation of *z*-scores, more significant differences were observed between the exposed and unexposed groups—the analysis of the data indicated that the exposed group had lower respiratory function than the unexposed group. Table 4 provides a detailed summary of the *z*-scores for IOS, N_2_MBW, and DLCO. The results showed that the exposed group had significantly impaired results for R5-R20 (*P* = 0.029), LCI 2.5% (*P* = 0.002), and S_*acin*_ (*P* = 0.033) when compared to the unexposed group. Additionally, a noticeable tendency toward higher values for R5 (*P* = 0.059), AX (*P* = 0.051), and X5 (*P* = 0.054) was observed in the exposed group.

**TABLE 4:** Lung function tests *z*-scores for exposed vs. unexposed in IOS (*n* = 15 vs. 15), N_2_MBW (*n* = 15 vs. 14), and DLCO (*n* = 13 vs. 14).

Variable	Unexposed Median (min–max)	Exposed Median (min–max)	*P*-value
R5 *z*	0.60 (−0.55 to 1.81)	1.48 (−0.89 to 13.16)	0.059
R20 *z*	0.10 (−0.23 to 0.95)	0.61 (−1.09 to 4.69)	0.382
R5-R20 *z*	0.29 (−0.63 to 3.28)	2.00 (−0.23 to 16.70)	0.018
X5 *z*	−0.63 (−1.15 to 0.54)	−1.12 (−6.53 to 0.05)	0.054
AX *z*	0.42 (−0.49 to 6.24)	2.94 (−0.72 to 59.61)	0.051
LCI 2.5% *z*	1.65 (−0.92 to 5.14)	3.64 (−0.63 to 27.55)	0.002
S_*cond*_ *z*	1.63 (−3.15 to 17.56)	4.26 (−3.67 to 14.90)	0.201
S_*acin*_ *z*	−0.14 (−1.99 to 3.03)	2.42 (−0.87 to 20.67)	0.033
DLCO *z*	0.58 (−0.13 to 1.91)	0.65 (−2.30 to 1.45)	0.479
DLCO/VA *z*	0.90 (−0.56 to 2.53)	1.12 (−0.73 to 2.64)	0.840
VA *z*	−0.6 (−3.06 to 1.84)	−0.98 (−3.12 to 3.07)	0.264

Data are reported as and *z*-scores (*z*). Median (min–max). IOS, impulse oscillometry, AX, area of reactance; DLCO, diffusing capacity for carbon monoxide; R5-R20, the difference between resistance at 5 and 20 Hz; LCI 2.5%, lung clearance index; N_2_MBW, nitrogen multiple breath washout; R5, Resistance at 5 Hz; R20, resistance at 20 Hz; S_*cond*_, S_*acin*_, ventilation heterogeneity in the conducting and acinar airways, respectively; VA, alveolar volume; X5, reactance at 5 Hz. We applied the Mann–Whitney U test to determine the differences between the exposed and unexposed groups. We set the significance level at *P* < 0.05.

**FIGURE 9:**
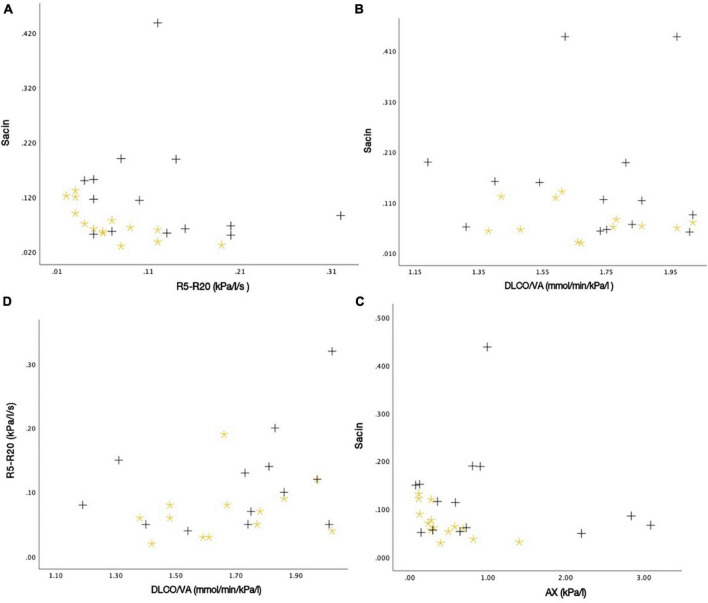
The associations between different measures of lung function as scatter plots: **(A)** S_*acin*_ and R5-R20 (*r* = –0.082, *P*-value = 0.678), **(B)** S_*acin*_ and DLCO/VA (*r* = –0.023, *P*-value = 0.810), **(C)** S_*acin*_ and AX (*r* = –0.045, *P*-value = 0.820), and **(D)** R5-R20 and DLCO/VA (*r* = 0.324, *P*-value = 0.106). Black plus = SM-exposed participant. Yellow star = Unexposed participant.

After analyzing the scatter plots, no statistically significant relationships were observed between the different measures of lung function with IOS, N_2_MBW, and DLCO (Figure 9). For instance, a very weak negative correlation was found between S_*acin*_ and R5-R20 (*P* = 0.68). Similarly, the association between S_*acin*_ and DLCO/VA had a *P*-value of 0.81, which was not statistically significant. Another weak negative correlation was observed between S_*acin*_ and AX, with a *P*-value of 0.82. However, this correlation was not statistically significant. Lastly, R5-R20 and DLCO/VA had a moderate positive correlation, with a *P*-value of 0.11. Nevertheless, this correlation was not statistically significant.

## 4 Discussion

Individuals exposed to SM can experience serious, long-lasting respiratory problems that gradually worsen over time, potentially leading to disabilities. This study is among the first to investigate the small airway function in SM-exposed individuals using non-traditional noninvasive PFTs, e.g., ISO and N_2_MBW, compared to unexposed control individuals. The findings revealed a positive association between exposure to SM and SAD. The exposed group had significantly higher respiratory resistance, stiffness, and ventilation heterogeneity in the small airways than the unexposed group.

Impulse oscillometry and N_2_MBW showed small airway dysfunction in the SM-exposed group, with statistically significant differences in the absolute measured values and abnormal *z*-scores for variables related to peripheral small airways compared to none in the unexposed group. In addition, the method was validated on individual levels; 8 of the 15 exposed participants who reported dyspnea upon walking had abnormal results for S_*acin*_, LCI 2.5%, R5-R20, and AX.

Impulse oscillometry devices revealed that 8 of the 15 exposed participants had abnormal values for R5-R20, suggesting higher respiratory resistance in small airways, and 10 had abnormal values for AX, revealing stiffness in small airway. This contrasts with the unexposed group, where only four participants had abnormal values for AX. In addition, five participants in the exposed group had abnormal values for R5 indicators of total airway resistance, and two had abnormal values for R20 predicting central airway resistance compared to none in the unexposed group, indicating that the exposed group had more impaired respiratory function in large and central part of airways in addition to small airways. These findings are consistent with those of other studies, indicating that exposure to SM can cause significant damage to the central airways, as detected through traditional techniques such as spirometry and HRCT (11, 12, 49).

Concerning the N_2_MBW test, the variation within the exposed group was large, with a few individuals showing large deviations, possibly due to different exposure levels and susceptibility; Eight participants in the exposed group and three participants in the unexposed group had abnormal S_*acin*_ values (Figure 5). For LCI 2.5%, the overlap was bigger: 14 exposed and 5 unexposed participants had values above the reference level (*z*-scores of +1.96) (Table 3). However, we cannot exclude the possibility that other factors may also have played a role; for instance, the individual in the unexposed group also exhibited abnormal values.

Among the IOS variables, R5-R20 is presumably more specific for peripheral airway resistance; however, it does not reflect the pathology of the smallest airways. AX measures the reactance in the small airways, that is, the elastance or stiffness of the airways. In comparison, S_*acin*_ detects ventilation heterogeneity in the very distal/acinar airways. Consequently, these parameters are considered complementary for the identification of SAD. The current study found that all these measures may be influenced; however, they may show different patterns in different individuals, suggesting that exposure to SM may have different long-term consequences on small airways, possibly reflecting different SM exposure situations and different inherent susceptibilities. However, other factors may have also influenced the results, as a long time had passed since the SM exposure.

The results of DLCO in this study are consistent with those of a previous study that included 23 SM-exposed patients with abnormal spirometry, HRCT, and bronchoscopy results but normal DLCO when assessed almost 14 years after SM exposure (17). The large alveolar surface and large reserve capacity for gas diffusion at rest might explain this. Thus, DLCO is not considered a useful method, especially to assess SAD in SM-exposed individuals.

The abnormal values for R20 and R5, LCI 2.5%, and S_*cond*_ suggest that lung function impairment may extend in other areas of the lung beyond the small airways. This finding aligns with that of other studies, which demonstrated bronchiectasis, atelectasis, dilated central airways, bronchial wall thickening, and interlobular septal wall thickening in individuals exposed to SM (11, 12, 49).

With IOS and N_2_MBW peripheral small airways related variables, at least one abnormal value was revealed for 93% of the exposed participants compared to 53% in the unexposed group (Figure 5); these individuals with abnormal values in the exposed group had clinical manifestation in terms of dyspnea upon walking on smooth ground, but not in the unexposed one. The reasons for the abnormal values in the unexposed participants may vary. The inclusion criteria for the control group did not consider other potential sources of exposure, such as environmental pollutants, second-hand smoking, occupational hazards, or other diseases that could potentially impact lung function. Additionally, the use of local Swedish references (24) in the study may have narrowed the reference range, resulting in abnormal values for both the control and exposed groups. Moreover, the prevalence of undiagnosed asthma and COPD in the population is worth noting (50, 51). However, one unexposed participant with abnormal S_*acin*_ was later diagnosed with lung disease by their family physician. It is worth noting that the unexposed participants with abnormal *z*-scores did not report respiratory clinical symptoms contrary to the exposed participants. The existence of abnormal values in unexposed control in our article is consistent with the results of a previous study that found both pulmonary symptoms and abnormal spirometry findings in the unexposed control group compared to SM-exposed individuals (52).

The current study’s results, suggesting SAD, align with those of other studies conducted on SM-exposed participants 10–15 years following SM exposure. Previous studies reported proinflammatory and inflammatory mediators in the bronchoalveolar lavage fluid, such as neutrophils, indicating injury to the bronchiole tree and a high incidence of bronchiole obliterans in SM-exposed survivors (11, 16). Exposure to SM may induce chronic inflammation in the airways by the accumulation of inflammatory cells that release proinflammatory substances, such as cyclooxygenase-2 and 12-lipoxygenase, and reduce the production of protective substances, including surfactants (18, 53–55). These pathological processes may successively deteriorate and result in small airway scarring that is undetectable by conventional imaging and spirometry tests in the early stages.

Furthermore, the results of the present study are significant as they indicate that certain SM-exposed participants had abnormal indices linked to small airways, even though they showed no clinical symptoms. This finding is consistent with that of a previous study, which identified signs of air trapping on HRCT in asymptomatic individuals exposed to SM (26). These findings suggest that the effects of SM exposure on lung function may not be immediately apparent, emphasizing the need to monitor for any potential respiratory issues that could develop in high-risk individuals who were exposed to SM (9).

Fres, another indicator in IOS, is the frequency at which the airways and lung tissues vibrate smoothly during breathing at the normal interval reference (7–12 Hz) reported in a previous study (42). With a score range (8–42) and a mean value (18.5), we noticed a considerable difference (*P* = 0.057) in the fres values between the exposed and unexposed groups in our study population (as shown in Figure 4). This indicates impacted airways among the exposed group. Unfortunately, we could not calculate the *z*-score for fres as we could not access a reference.

Previous studies have revealed a mixed picture of the lung status in SM-exposed participants, which may be attributed to the diagnostic techniques used (56). In a study of 15 Iranian SM-exposed survivors with respiratory symptoms, 13 had normal spirometry tests, 1 had mild restriction, and 1 had an obstructive pattern 20 years post-exposure. Histological biopsy analysis revealed that 50% of these patients had either bronchiolitis obliterans or signs indicative of its presence (10). This is in line with the results of our study, where 53% of the SM-exposed group had heterogeneity in S_*acin*_ and higher resistance in small airways (R5-R20) and 66% showed stiffness in small airways (AX). Hence, IOS and N_2_MBW as small airway-sensitive tests in SM-exposed lung investigations might help clarify the structural and parenchymal pulmonary pathological changes. The current study, which revealed the presence of SAD in SM-exposed participants, might provide a plausible explanation for the gradual manifestation of clinical respiratory symptoms in SM-exposed survivors (9). Small airways are the quiet zone of the airways; thus, SAD can often remain undetected by spirometry until the manifestation of significant clinical symptoms (10, 23). Furthermore, the presence of SAD may be important when assessing the responses to cortisone and bronchodilator inhalation in SM-exposed patients. Traditional inhalers may not adequately reach damaged peripheral airways owing to the powder’s particle size (57, 58).

The SAD verified by biopsy in SM-exposed individuals despite normal spirometry and HRCT results (27, 59) highlights the limitations of HRCT and spirometry in detecting the existence of SAD in the early stages among SM-exposed patients. While expiratory CT air trapping is a reliable indicator of SAD, visually detecting diffuse air trapping can be challenging (59). Spirometry tests also require a good technique and the patient to maneuver. Moreover, it registers SAD only after obstruction of a significant portion of the small airways (27, 28). Thus, early detection of SAD is crucial for controlling damage to small airways (26). Noninvasive methods, such as IOS and N_2_MBW, are easy to perform, independent of patient effort, register modifications in small airways long before spirometry (60), and lack radiation risk as in HRCT with repeated application. Moreover, IOS could prove useful particularly in SM-exposed patients who are unable to undergo spirometry tests. Additionally, these methods may be useful in providing complementary information to spirometry tests and HRCT for monitoring SAD and evaluating therapeutic intervention in SM-exposed patients (61).

### 4.1 Limitations and strengths

This study, while providing valuable insights into SAD in individuals exposed to SM, has certain limitations. Firstly, its generalizability to larger populations is limited owing to the small sample size; moreover, a selection bias could exist as participants with residual respiratory symptoms might have been more inclined to participate. Our findings illustrate the importance of including measurements of SAD in individuals with residual respiratory symptoms after SM exposure. Secondly, the study’s observational nature makes it difficult to comment on the causality between SM exposure and the outcomes. A long time had elapsed since the exposure, and many other exposures may also have affected the respiratory system. Moreover, we have limited data on the extent of exposure, which varied in the SM-exposed group.

Another limitation might be the lack of spirometry tests and imaging data. The initial study protocol included spirometry; however, most participants could not undergo repeated spirometry tests with adequate quality, leading to the exclusion of spirometry tests from the 2022 study protocol. Overall, this could be attributed to a combination of participant-related factors (lack of motivation or language barrier), and technical issues (limited resources). Comparing the results of physiological lung function tests with imaging would be highly interesting; however, this was impossible owing to limited resources.

Despite these limitations, the strength of this study is that it is one of the first to investigate SAD in SM-exposed participants vs. an unexposed control group and compare different non-traditional noninvasive lung physiological diagnostic techniques. IOS and N_2_MBW are noninvasive PFTs that can detect SAD in survivors long after exposure. The absolute measured values and *z*-scores were highly consistent in evaluating abnormal values, which strengthened the outcomes of this study. The existence of abnormal values among unexposed controls indicate that some of controls may have been sick too, thus the significance of the findings by this study may have been underestimated.

In summary, no notable correlations were observed between the small airways indicators, IOS, N_2_MBW, and DLCO. Of the three, IOS is the most precise, N_2_MBW is sensitive but has some limitations in the specificity, and DLCO exhibits low sensitivity and specificity (Table 3). Ultimately, the IOS and N_2_MBW are highly valuable in detecting SAD in the early stage among SM-exposed individuals, providing additional information to that obtained using traditional methods for monitoring SAD, and evaluating therapeutic interventions for patients exposed to SM.

However, neither IOS nor N_2_MBW is the gold standard for evaluating small airways. Nevertheless, further assessment of SAD with IOS and N_2_MBW in a larger population of SM-exposed participants is warranted.

## 5 Conclusion

Exposure to SM was positively associated with long-term impairment of respiratory tract function in the small airways in 14 of 15 SM-exposed participants, of whom 8 individuals reported dyspnea during walking and 6 of 15 in the unexposed group with no reported respiratory clinical symptoms in the present study. Whether they are at risk of further deterioration and lung disease is unknown; however, exploring this in a large SM-exposed population would be of great interest. DLCO measurements suggest that SM is unlikely to significantly affect lung gas exchange at the alveolar-capillary interface in the long-term. Furthermore, both IOS and N_2_MBW should be employed to detect SAD in SM-exposed survivors as they provide complementary information. Identifying and characterizing the remaining pathology of the small airways in survivors of SM exposure is the first step toward improved treatment and follow-up.

## Data availability statement

The raw data supporting the conclusions of this article will be made available by the authors, without undue reservation.

## Ethics statement

The studies involving humans were approved by the Regional Ethical Review Board of Gothenburg, Sweden (2017-12-07599-17). The studies were conducted in accordance with the local legislation and institutional requirements. The participants provided their written informed consent to participate in this study.

## Author contributions

FM and A-CO conceived and designed the study. SK and FM prepared the materials and collected the data. FM conducted the statistical analysis in close collaboration with SK, which was then validated by YL. FM, AC-O, SK, and BD interpreted the data. FM wrote the initial draft of the manuscript. All authors reviewed and commented on various versions of the manuscript, read, and ultimately approved the final manuscript.
